# A cross-sectional national investigation of COVID-19 outbreaks in nurseries during rapid spread of the Alpha (B.1.1.7) variant of SARS-CoV-2 in England

**DOI:** 10.1186/s12889-022-14228-z

**Published:** 2022-10-02

**Authors:** Felicity Aiano, Kelsey McOwat, Chinelo Obi, Annabel A. Powell, Jessica Flood, Shivraj Bhardwaj, Kelly Stoker, Donna Haskins, Brian Wong, Marta Bertran, Maria Zavala, Johanna Bosowski, Samuel E. I. Jones, Zahin Amin-Chowdhury, Laura Coughlan, Mary Sinnathamby, Asad Zaidi, Rachel Merrick, Hongxin Zhao, Sharif Ismail, Mary E. Ramsay, Shamez N. Ladhani, Vanessa Saliba

**Affiliations:** 1 Immunisation and Vaccine Preventable Diseases Division, UK Health Security Agency, 61 Colindale Avenue, London, NW9 5EQ UK; 2 London School of Hygiene and Tropical Medicine, London, UK; 3 Paediatric Infectious Diseases Research Group, St. George’s University of London, London, UK

**Keywords:** SARS-CoV-2, Epidemiology, Children, Educational settings, Nurseries

## Abstract

**Background:**

In England, the emergence the more transmissible SARS-CoV-2 variant Alpha (B.1.1.7) led to a third national lockdown from December 2020, including restricted attendance at schools. Nurseries, however, remained fully open. COVID-19 outbreaks (≥ 2 laboratory-confirmed cases within 14 days) in nurseries were investigated to assess the risk of SARS-CoV-2 infection and cumulative incidence in staff and children over a three-month period when community SARS-CoV-2 infections rates were high and the Alpha variant was spreading rapidly across England.

**Methods:**

This was a cross-sectional national investigation of COVID-19 outbreaks in nurseries across England. Nurseries reporting a COVID-19 outbreak to PHE between November 2020 and January 2021 were requested to complete a questionnaire about their outbreak.

**Results:**

Three hundred and twenty-four nurseries, comprising 1% (324/32,852) of nurseries in England, reported a COVID-19 outbreak. Of the 315 (97%) nurseries contacted, 173 (55%) reported 1,657 SARS-CoV-2 cases, including 510 (31%) children and 1,147 (69%) staff. A child was the index case in 45 outbreaks (26%) and staff in 125 (72%) outbreaks. Overall, children had an incidence rate of 3.50% (95%CI, 3.21–3.81%) and was similar irrespective of whether the index case was a child (3.55%; 95%CI, 3.01–4.19%) or staff (3.44%; 95%CI, 3.10–3.82%). Among staff, cumulative incidence was lower if the index case was a child (26.28%; 95%CI, 23.54–29.21%%) compared to a staff member (32.98%; 95%CI, 31.19–34.82%), with the highest cumulative incidence when the index case was also a staff member (37.52%; 95%CI, 35.39–39.70%). Compared to November 2020, outbreak sizes and cumulative incidence was higher in January 2021, when the Alpha variant predominated. Nationally, SARS-CoV-2 infection rates in < 5 year-olds remained low and followed trends in older age-groups, increasing during December 2020 and declining thereafter.

**Conclusions:**

In this cross-sectional study of COVID-19 outbreaks in nurseries, one in three staff were affected compared to one in thirty children. There was some evidence of increased transmissibility and higher cumulative incidence associated with the Alpha variant, highlighting the importance of maintaining a low level of community infections.

**Supplementary Information:**

The online version contains supplementary material available at 10.1186/s12889-022-14228-z.

## Background

The rapid spread of SARS-CoV-2, the virus responsible for COVID-19, forced many countries to impose national lockdowns [1], including closure of educational settings [2]. In England, the first imported cases of SARS-CoV-2 were identified in January 2020 and increased rapidly in early March, leading to school closures on 20 March and nationwide lockdown from 23 March 2020 [3]. Cases increased until mid-April, then declined in subsequent weeks and remained low across all age-groups during the summer months [3, 4], allowing full attendance for all children into educational settings in September 2020 [4].

Compared to adults, children have been reported to have a lower risk of symptomatic disease, hospitalisation or death [3, 5, 6]. The vast majority of children infected with SARS-CoV-2 remain asymptomatic or develop a mild, transient illness [6, 7]. Whilst the benefits of children returning to school on their educational attainment, physical, mental, emotional and social well-being cannot be denied [8–11], the full reopening of schools raised the potential for widespread SARS-CoV-2 transmission in educational settings [12], with possible spill-over into households of students and staff and the wider community, or vice-versa [13].

From mid-August 2020 and prior to schools reopening to all students, SARS-CoV-2 infections started increasing nationally, first in adults and then in children, leading to a second national lockdown during November 2020, whilst keeping educational settings fully open. SARS-CoV-2 cases subsequently declined, first in adults and then in school-aged children [4]. The emergence of a novel, more transmissible variant of concern (Alpha, VOC-202012/01 or B.1.1.7), however, was associated with rapid increases in SARS-CoV-2 infection rates across England from late November 2020 [14], leading to a third national lockdown in January 2021, which included restricted primary and secondary school attendance for children of keyworkers and vulnerable children [4]. Nurseries, however, remained open to all children because the risk of SARS-CoV-2 infection and transmission in infants and toddlers was considered to be very low [4, 15].

Compared to primary and secondary schools, nurseries provide a different challenge from an infection control perspective. The premises are often much smaller, with a higher staff-to-child ratio, and the very young age of the children makes physical distancing from staff unfeasible [16, 17]. Public Health England (PHE), therefore, initiated rapid national investigation of COVID-19 outbreaks in nurseries to assess the risk of SARS-CoV-2 infection and cumulative incidence in staff and children over a three-month period when community SARS-CoV-2 infections rates were high and the Alpha variant was spreading rapidly across England [18]. We also compared trends in SARS-CoV-2 infections rates in nurseries with national surveillance data for children and adults in England.

## Methods

In England, educational settings are regularly provided with updated guidance on infection control measures, including recommendations for good ventilation, regular hand-sanitising, social distancing, and enhanced cleaning; face masks and face coverings are not recommended for staff or children in nurseries [19, 20]. All individuals, including staff and children, have access to free community SARS-CoV-2 RT-PCR testing if they develop COVID-19 symptoms (fever, new continuous cough, loss of test/smell). Cases and their close contacts must isolate for 10 days. Outbreaks reported to PHE are routinely recorded in HPZone [21], an online national case management system used by local health protection teams (HPTs) to record events that require public health management.

### Outbreak investigation

We used the same methodology to investigate COVID-19 outbreaks in nurseries as for primary and secondary school outbreaks, defined as “ ≥ 2 laboratory-confirmed cases within 14 days” [22]. “Nurseries”, also known as early years settings, includes all non-domestic settings which offer care for children from birth to 5 years, after which time they start formal education in school. Childminders and babysitters were not included. Briefly, because HPZone contains very limited information on individual outbreaks, nurseries reporting a COVID-19 outbreak between 02 November 2020 and 31 January 2021 were contacted between 08 and 23 February 2021 to provide detailed information about the outbreak, either by telephone or completing an online questionnaire using SnapSurvey v.11. Collected information included the size and structure of bubbles (defined as a group of staff and children who performed all activities together and did not interact with other bubbles) and the degree of contact between staff with other staff and with children. Staff were considered to have “direct contact” with children if they had a childminding or other roles requiring close contact with children. Those in other roles, such as administration, were considered to have “no direct contact” with children.

### Data analysis

Data were analysed using R studio (version 1.3.1056) and Stata v.15.1 (Statacorp, Tx), and are mainly descriptive. The start of an outbreak was taken as the symptom onset date in the index PCR-confirmed case or, for asymptomatic cases, their date of PCR sample. Denominators for staff and children by age-group and bubble were reported by individual nurseries. Denominators for the number of nurseries and educational settings in England were estimated using the total number of possible settings for different age-groups. A setting with a combination of nurseries, primary or secondary schools was counted separately for each setting (Source: Department for Education) [23]. The proportion of cases due to the Alpha variant in England was obtained from national surveillance reports [24]. We assessed the impact of the Alpha variant by analysing changes in the number and size of nursery outbreaks as well as case numbers and cumulative incidence in staff and children during November 2020 (when the Alpha variant was responsible for < 20% of confirmed SARS-CoV-2 infections in England), December 2020 (20–80%) and January 2021 (> 80%). Non-normal continuous data are described as medians with interquartile ranges. Proportions were compared using the chi-squared or Fisher’s Exact test. Cumulative incidence was calculated by dividing the number of cases by the population under investigation and compared using Fisher’s Exact probability tests via the prop.test function in R studio (version 1.3.1056) [25]. Cumulative incidence was compared by source of index case. Analyses were not adjusted for multiple comparisons.

### Ethical review and informed consent

PHE has legal permission, provided by Regulation 3 of The Health Service (Control of Patient Information) Regulations 2002, to process patient confidential information for national surveillance of communicable diseases and as such, individual patient consent and approval from an ethics committee is not required.

## Results

**Fig. 1:**
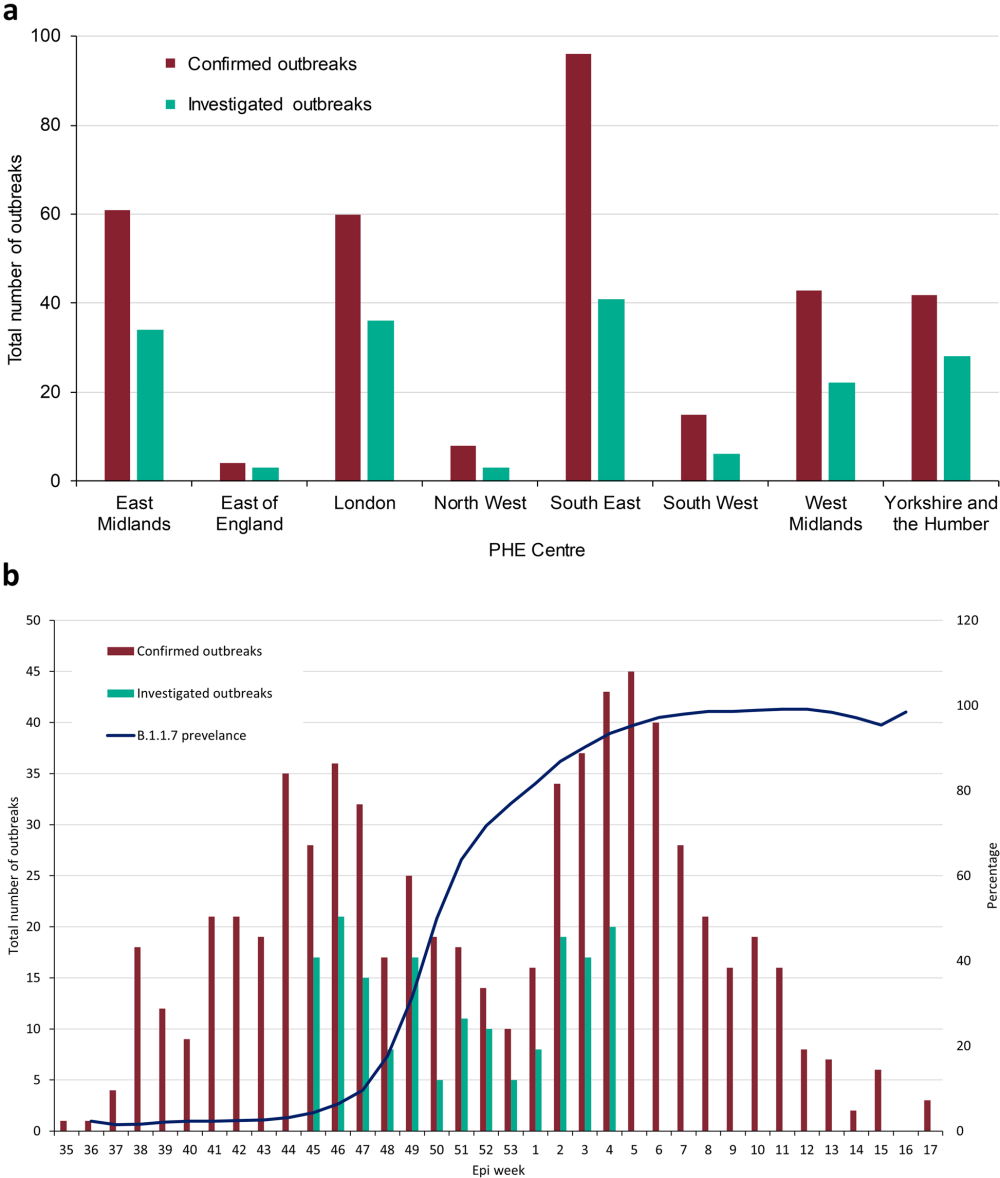
Total number of outbreaks in England that were reported to Public Health England (PHE) between 02 November 2020 and 31 January 2021 (**a**) by PHE Centre and those included in the PHE investigation and (**b**) by epi week, with proportion of confirmed SARS-CoV-2 infections in the community that were due to the ALPHA (B.1.1.7). variant

Between 02 November 2020 (week 45) and 31 January 2021 (week 4), 324 nurseries reported a COVID-19 outbreak to PHE, comprising 1.0% (324/32,852) of nurseries across England (Fig. 1a). The number of outbreaks peaked in early November, then declined until end of December before increasing again in January and declining again from mid-February to the end of April 2021. (Fig. 1b).

Between 09 and 23 February 2021, 315 (97%) nurseries were contacted and 181 (57%) completed the survey. Eight settings provided insufficient information; therefore, 173 nurseries from all English regions were included in the analysis (Fig. 1a). There were 1,657 SARS-CoV-2 cases, including 510 (31%) children and 1,147 (69%) staff linked to these outbreaks. A median of 8 (IQR: 5–13; range 2–33) individuals were affected per outbreak (Table 1). Nurseries reporting an outbreak organised their children and staff into anywhere between 1–10 bubbles. Of the 171 that reported their bubble setup, 42% (72/171) of outbreaks occurred across > 3 bubbles, 33% (56/171) involved two bubbles and 25% (43/171) involved one bubble (Supplement Table S1). Overall, 99 settings closed at least once because of the outbreak (4 closed twice, 1 closed 3 times), 96 of which reported how long they closed for: up to one week (28 nurseries), 7–13 days (33 nurseries) or 14–28 days (35 nurseries). A child was the index case in 45 outbreaks (45/173, 26%) and a staff member in 125 (125/173, 72%) (Table 2). Having COVID-19 symptoms was the most common reason for testing in the first four cases of each outbreak (411/603, 68%), followed by being a contact of a nursery case (92/603, 15%) and being a contact of a household case (58/603, 10%) (Supplement Table S4). There were 28 hospitalisations, including one child, and no deaths.

**Table 1:** Summary of nursery outbreaks in England that were reported to Public Health England (PHE) between 02 November 2020 and 31 January 2021

	**Month**			**Overall**
	**November 2020**	**December 2020**	**January 2021**	
**Number of outbreaks**	64 (37%)	43 (25%)	66 (38%)	173 (100%)
**Median (IQR) cases per outbreak**	5 (3–9)	11 (6.5–15.5)	10 (6–15)	8 (5–13)
**Mode (range) of cases per outbreak**	3 (2–20)	5 (2–31)	6 (2–33)	2 (2–33)
**Cases in children**	115 (27%)	146 (30%)	249 (34%)	510 (31%)
Cumulative incidence **in children (95% confidence interval)**	2.34% (1.94–2.81)	3.89% (3.30–4.57)	4.21% (3.72–4.77)	3.50% (3.21–3.81)
**Cases in staff with direct contact with children**	290 (68%)	309 (63%)	429 (58%)	1028 (62%)
Cumulative incidence **in staff with direct contact with children (95% CI)**	26.58% (24.00–29.33)	37.87% (34.45–41.31)	35.34% (32.66–38.11)	32.94% (31.30–34.62)
**Cases in staff without contact with children**	23 (5%)	36 (7%)	60 (8%)	119 (7%)
Cumulative incidence **in staff without contact with children (95% CI)**	11.56% (7.62–17.03)	21.95% (16.03–29.22)	26.55% (21.02–32.9)	20.20% (17.08–23.72)
**Cases in all staff**	313 (73%)	345 (70%)	489 (66%)	1147 (69%)
Cumulative incidence **in all staff (%)**	24.26% (21.97–26.72)	35.20% (32.23–38.30)	33.96% (31.52–36.48)	30.92% (29.44–32.44)
**Total cases**	428 (26%)	491 (30%)	738 (45%)	1657 (100%)
**Total c**umulative incidence **%**	6.90% (6.29–7.56)	10.36% (9.52–11.27)	10.04% (9.37–10.76)	9.06% (8.65–9.49)

**Table 2:** Number of SARS-CoV-2 cases and cumulative incidence among staff and students by index case in nurseries reporting an outbreak to Public Health England (PHE) between 02 November 2020 and 31 January 2021

	**When the Index Case is:**			
	**A child**	**Staff member with direct contact with children**	**Staff member swith no contact with children**	**All staff members**
Number of outbreaks	45	117	8	125
Number of children	3971	9434	850	10,284
Cases in children	141	317	37	354
Cumulative incidence (95% confidence interval)	3.55% (3.01–4.19)	3.36% (3.01–3.75)	4.35% (3.12–6.01)	3.44% (3.10–3.82)
Number of staff with direct contact with children	848	1983	211	2194
Cases in staff with direct contact with children	214	744	48	792
Cumulative incidence (95% confidence interval)	25.24% (22.37–28.33)	37.52% (35.39–39.70)	22.75% (17.40–29.12)	36.10% (34.09–38.15)
Number of staff with no contact with children	111	399	48	447
Cases in staff with no contact with children	38	61	18	79
Cumulative incidence (95% confidence interval)	34.23% (25.66–43.92)	15.29% (11.98–19.28)	37.50% (24.32–52.67)	17.67% (14.31–21.60)
Total number of staff	959	2382	259	2641
Total cases staff	252	805	66	871
Cumulative incidence (95% confidence interval)	26.28% (23.54–29.21)	33.80% (31.90–35.74)	25.48% (20.38–31.32)	32.98% (31.19–34.82)
Total (staff and children)	4930	11,816	1109	12,925
Total cases	393	1122	103	1225
Cumulative incidence (95% confidence interval)	7.97% (7.24–8.77)	9.50% (8.98–10.04)	9.29% (7.68–11.19)	9.48% (8.98–10.00)

### Cumulative incidence in staff and students

**Fig. 2:**
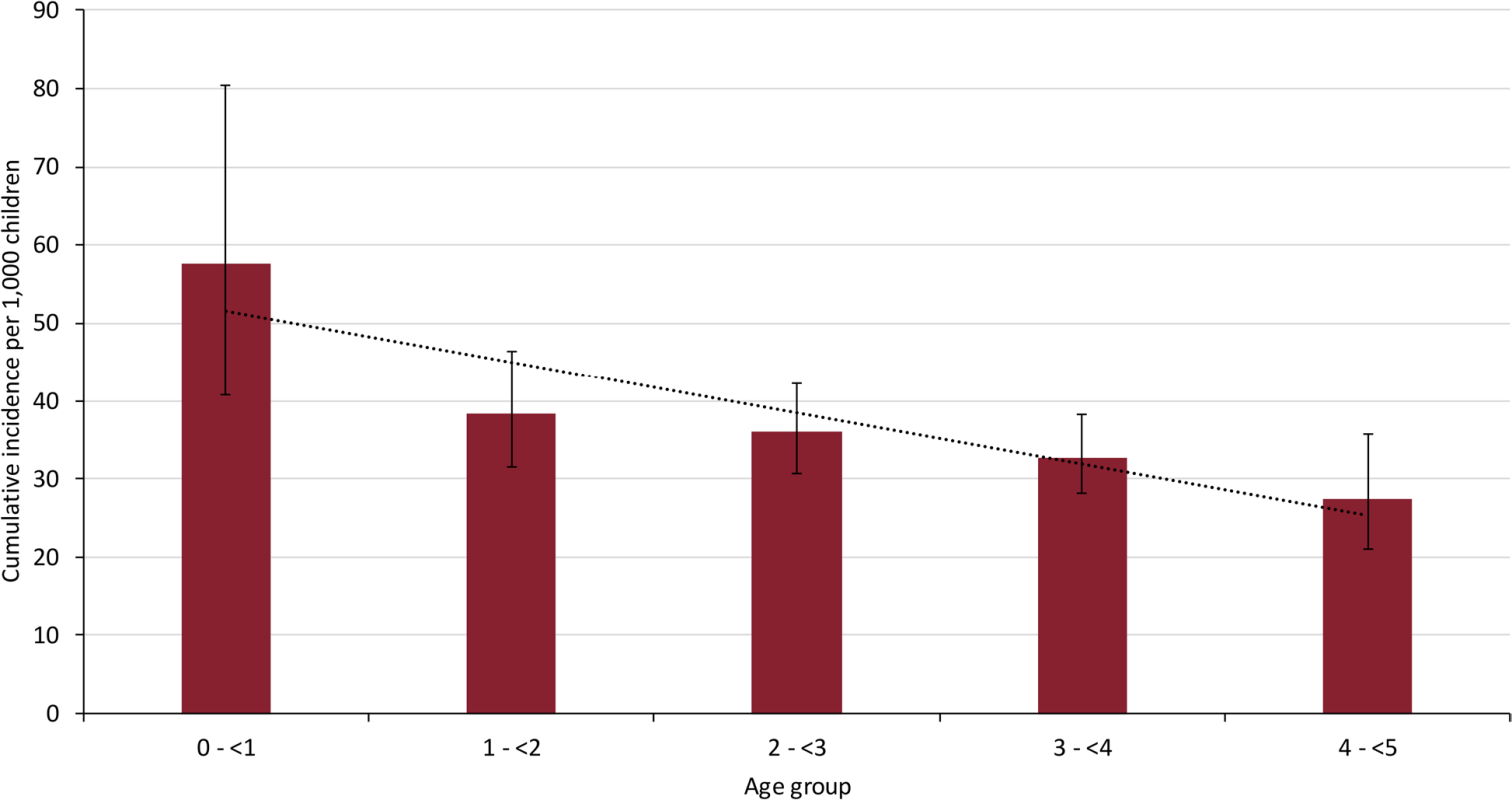
Cumulative incidence per 1,000 children attending a nursery in England that reported a COVID-19 outbreak between 02 November 2020 and 31 January 2021 and were subsequently investigated by Public Health England (PHE). The error bars indicate 95% Confidence Intervals

Overall, children had an cumulative incidence of 3.50% (95%CI, 3.21–3.81%) compared to 30.92% (95%CI, 29.44–32.44%) among staff (Table 1). In children, SARS-CoV-2 cumulative incidence was highest in the < 1 year-olds (5.76%; 95%CI, 4.08–8.04%) and decreased with age, with 4 year-olds having the lowest cumulative incidence (2.75%; 95%CI, 2.10–3.58%) (Fig. 2). Staff with direct contact with children had the highest cumulative incidence (32.94%; 95%CI, 31.30–34.62%), followed by staff with no direct contact (20.20%, 95% CI, 17.08–23.72%) (Table 1).

Cumulative incidence was lowest in children, irrespective of whether the index case was a child (3.55%; 95%CI, 3.01–4.19%) or a staff member (3.44%; 95%CI, 3.10–3.82%). Among staff, overall, cumulative incidence was lower if the index case was a child (26.28%; 95%CI, 23.54–29.21%%) compared to a staff member (32.98%; 95%CI, 31.19–34.82%), with the highest cumulative incidence among direct staff when the index case was also a direct staff member (37.52%; 95%CI, 35.39–39.70%) (Table 2). Compared to November 2020, when there was little transmission of the Alpha variant in England, the number of cases, median and mode number of individuals affected per outbreak and cumulative incidence in children and staff was all higher in January 2021, when nearly all cases were due to the Alpha variant nationally, while the number of reported outbreaks were similar (64 vs. 66) for these two months (Table 1).

### Facilities, social distancing and infection control measures

Nurseries experiencing a COVID-19 outbreak reported that 49% (85/172) of their staff members were able to maintain physical distancing between each other most of the time throughout the day, while 39.5% (68/172) reported social distancing “rarely”, “never” or “some of the time”. Within individual bubbles, nurseries reported that 74% (127/172) of children were “never” able to maintain physical distancing with each other, and 64% (110/172) staff were “never” able to maintain physical distancing with children (Supplement Table S2). Across bubbles, 50% (83/166) of nurseries reported that their staff members were able to maintain physical distancing from staff in other bubbles all the time throughout the day, and 61% (102/166) of children were able to maintain physical distancing from children from other bubbles “all of the time” (Supplement Table S2).

Around two-thirds of nurseries had shared staff rooms (111/170, 65%) and 78% (131/169) had shared bathrooms used by staff members across different bubbles. Most settings had designated eating spaces (146/169, 86%) and bathrooms for children (112/168, 67%), assigned per bubble (Supplement Table S3). In 57% (99/173) of nurseries, staff were reported not to provide cross-cover across bubbles.

### National surveillance

**Fig. 3:**
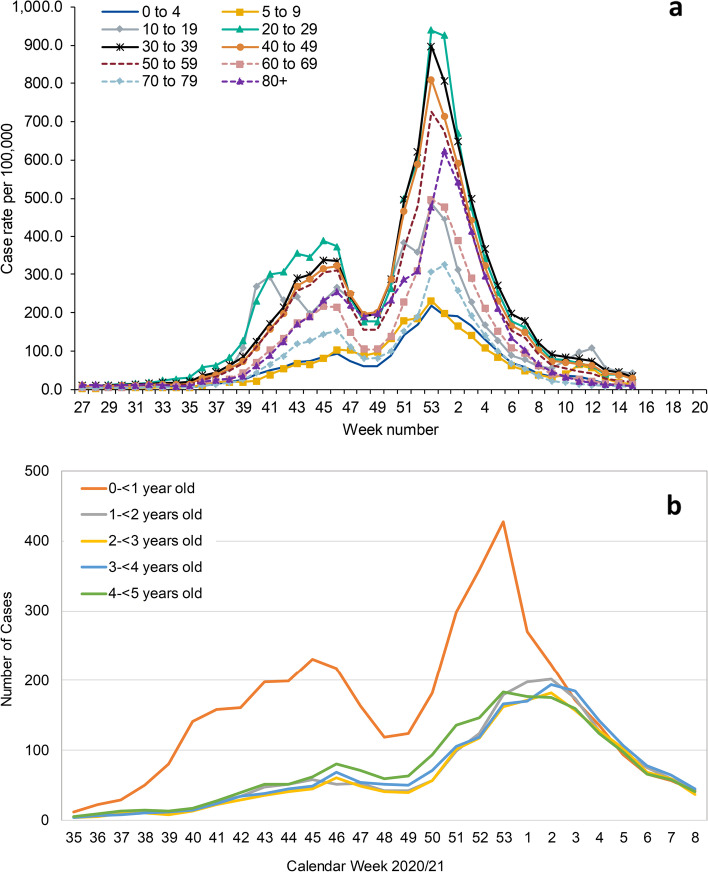
Weekly laboratory-confirmed SARS-CoV-2 infection rates per 100,000 in England (**a**) by age-group and (**b**) by age in years for children under 5 years of age

Nationally, weekly SARS-CoV-2 infection rates were lowest in children aged < 5 years and followed the same trends as the older age-groups (Fig. 3a and b). Thus, cases in children aged < 5 years increased slowly following the full reopening of all schools in September 2020 (week 36) and declined during the national lockdown in November 2020 when educational settings remained fully open to all students. Cases then increased from late November 2020, following the emergence of the Alpha variant in England until the end of 2020 before declining. In January and February 2021, when England was experiencing its third national lockdown, cases across all age-groups, including < 5 year-olds, continued to decline, at a time when nurseries remained open while primary and secondary schools had restricted attendance and in-person teaching for vulnerable children and children of key workers. Among < 5 year-olds, the highest weekly infection rates were in infants (< 1 year-olds) and declined with increasing age (Fig. 3a and b).

## Discussion

In England, 1% of nurseries reported a COVID-19 outbreak to PHE over a three-month period encompassing a rapid spread of the Alpha variant, which is associated with increased transmissibility [26], more severe disease and higher case fatality [27]. Children attending nurseries reporting an outbreak had a very low cumulative incidence compared to staff, irrespective of whether the index case was a child or staff. Consistent with community trends in < 5 year-olds, cumulative incidence was highest in infants and then declined with age. In contrast, infection rates were up to tenfold higher in staff, especially when the index case was another staff member. Nationally, although the Alpha variant was first identified in September 2020, the rapid spread due to this variant during December 2021 was responsible for a third national lockdown in England, which included restricted primary and secondary school attendance for children of keyworkers and vulnerable children in January 2021 [28]. Nationally, cases in < 5 year-olds remained low throughout the period and followed trends in older age-groups, increasing during December 2020 and then declining during January and February 2021. While the number of nursery outbreaks were similar in November 2020 and January 2021, we found some evidence of larger outbreak sizes and higher cumulative incidence in January 2021, when the Alpha variant predominated. Outbreaks reported in nurseries declined from mid-February to the end of April 2021.

Between 31 August and 18 October 2020, when all education settings were fully open in England, only 0.3% (87/32,852) nurseries reported a COVID-19 outbreak compared to 2% (450/18,943) primary schools and 10% (519/5,409) secondary schools. This compares with 1% (324/32,852), 4% (684/18,943) and 12% (646/5,409), respectively, between 02 November 2020 and 31 January 2021, which included a period when primary and secondary schools had restricted attendance for children of keyworkers and vulnerable children in January 2021. When outbreaks did occur in nurseries, however, cumulative incidence in staff (24.26%) and children (2.34%) during November 2020, when there was little circulation of the Alpha variant, was significantly higher than primary school teaching staff (9.81%), secondary school teaching staff (3.97%), secondary school students (1.20%) or primary school students (0.84%) during September and October 2020, although these estimates were generated during a period of different community prevalence (Fig. 3a) [22]. Others have also reported extensive transmission in individual nursery outbreaks, with high SARS-CoV-2 positivity rates and seeding of infection into households [29]. Although children are more likely than adults to be asymptomatic, serosurveys where antibody testing is used to capture both symptomatic and asymptomatic infections, have reported similar or lower seropositivity in children compared to staff members [30, 31], adding to the growing evidence that children – especially infants and toddlers – are not the main drivers of infection in the household, educational settings or the wider community [32]. In France, only 3.7% of 327 children and 6.8% of 197 staff in 22 day centres were seropositive for SARS-CoV-2 antibodies in June 2020, compared to 5.0% in non-clinical hospital staff controls [30]. Additionally, almost half the seropositive children (45%) had been in contact with an adult household member with confirmed COVID-19 [30], similar to our seroprevalence study in primary schools where most children were infected at home [31].

Among children attending nurseries reporting a COVID-19 outbreak, the highest incidence was in infants and then declined with age, which was also observed in the national surveillance data [3]. A likely explanation is that it is not possible to maintain social distancing or strict infection control between the younger infants and staff or parents because they require frequent and prolonged close contact for feeding and self-care. This was also frequently reported by nurseries participating in our investigations, along with difficulties in maintaining physical distancing between staff both within and across bubbles, which was compounded by sharing of staff rooms and bathrooms by staff across different bubbles in many nurseries. These factors likely contributed to the high cumulative incidence among staff, especially when the index case was also a staff member.

### Limitations

Not all outbreaks would have been reported to PHE, as smaller, less complex outbreaks would have been managed by the settings themselves, with support from the National Schools Advice line and other partners such as Local Authorities. Additionally, HPTs in some regions with high COVID-19 incidence only recorded larger outbreaks with at least 5 cases, complex outbreaks or those requiring public health action. We also relied on the settings to report cases among staff and children and, since the outbreaks were not investigated with mass testing for SARS-Cov-2 infection, it is possible that the primary case may have been an asymptomatic child or adult who remained undetected. We are also unable to comment on the extent of asymptomatic spread in the affected settings for the same reason. In a recent Polish nursery outbreak, for example, wider RT-PCR testing of nursery staff, children and family members found that most of those infected with SARS-CoV-2 were asymptomatic [29]. Another limitation is that the lack of viral genome sequencing data precludes assessment of transmission since it is not possible to determine whether the outbreaks were due to widespread transmission of a single strain or multiple introductions of different virus strains into nurseries [15]. A higher community SARS-CoV-2 infection rate, for example, would result in more opportunities for virus introduction into nurseries and, therefore, higher estimates of cumulative incidence. Additionally, the lack of genome sequencing data meant that we were only able to assess the potential impact of Alpha indirectly by comparing outbreak size and cumulative incidence over time. We were unable to assess the nurseries who did not complete the survey. As such, this may have biased the results as those with time and capacity to complete the questionnaire may have had some systematic differences, such as staffing numbers, which could have affected the results. Finally, we were unable to objectively assess or compare adherence to physical distancing and other infection control measures adopted by the nurseries.

## Implications and conclusions

In England, COVID-19 outbreaks were rare in nurseries and cumulative incidence was low in children but substantially higher among staff members, higher than in primary or secondary schools [22]. In addition to the difficulties in physical distancing from children frequenting nurseries, other potential contributors for transmission among staff included shared use of staff rooms and bathrooms. This has important implications not only for SARS-CoV-2 but also for other highly-transmissible viruses such as influenza and noroviruses, highlighting the importance of promoting and maintaining rigorous infection control practices at all times and a need for better physical structuring of nurseries as we aim to build back better from the current pandemic. Additionally, we have previously reported a strong correlation between community SARS-CoV-2 infection rates and risk of outbreaks in educational settings [33], highlighting the importance of maintaining low community infection rates to reduce the risk of introducing the virus into nurseries and other similar childcare and educational settings. Finally, every effort should be made to promote vaccination – not only against COVID-19 but also against other vaccine-preventable infections – among nursery staff. Further studies are needed to assess whether outbreaks in educational settings are due to multiple introductions or widespread transmission of a single strain, and the impact of novel variants in educational settings.

## Supplementary Information

**Additional file 1: Table S1.** Size and number of bubbles in nurseries reporting a COVID-19 outbreak to Public Health England (PHE). **Table S2.** Reported social distancing between and within bubbles in nurseries reporting a COVID-19 outbreak to Public Health England (PHE). **Table S3.** Shared facilities in nurseries reporting a COVID-19 outbreak to Public Health England (PHE). **Table S4.** reason for testing in the first four cases, where known, among staff and children in nurseries reporting a COVID-19 outbreak to Public Health England (PHE).

## Data Availability

The datasets used and/or analysed during the current study are available from the corresponding author on reasonable request. Applications for relevant anonymised data should be submitted to the UK Health Security Agency Office for Data Release.

## Abbreviations

PHE: Public Health England
RT-PCR: Real time polymerase chain reaction
UKHSA: UK Health Security Agency
