# Supplementary material for: A cross-sectional national investigation of COVID-19 outbreaks in nurseries during rapid spread of the Alpha (B.1.1.7) variant of SARS-CoV-2 in England
Source: BMC Public Health. 2022 Oct 2;22:1845. doi: 10.1186/s12889-022-14228-z (PMC9526524; doi:10.1186/s12889-022-14228-z)
Supplement: Supplementary file 1 — Additional file 1: Table S1. Size and number of bubbles in nurseries reporting a COVID-19 outbreak to Public Health England (PHE). Table S2. Reported social distancing between and within bubbles in nurseries reporting a COVID-19 outbreak to Public Health England (PHE). Table S3. Shared facilities in nurseries reporting a COVID-19 outbreak to Public Health England (PHE). Table S4. reason for testing in the first four cases, where known, among staff and children in nurseries reporting a COVID-19 outbreak to Public Health England (PHE). [file 12889_2022_14228_MOESM1_ESM.docx]

**Assessment of COVID-19 outbreaks in nurseries during rapid spread of the Alpha (B.1.1.7) variant of SARS-CoV-2 in England**

**Felicity Aiano, MSc,^1^ Kelsey McOwat, MSc,^1^ Chinelo Obi, MSc,^1^ Annabel A Powell, BSc,^1^ Jessica Flood, PhD,^1^ Shivraj Bhardwaj, MSc,^1^ Kelly Stoker, MSc,^1^ Donna Haskins, MSc,^1^ Brian Wong, BSc^1^ Marta Bertran, MSc, ^1^ Maria Zavala, MSc,^1^ Johanna Bosowski, MSc,^1^ Samuel Jones, PhD,^1^ Zahin Amin-Chowdhury, BSc,^1^ Laura Coughlan,^1^ Mary Sinnathamby, MPH^1^, Asad Zaidi, MSc,^1^ Rachel Merrick, MSc,^1^ Hongxin Zhao, PhD, ^1^ Sharif Ismail, MBBS,^1,2^, Mary E Ramsay, FFPH,^1,2^ Shamez N Ladhani, MRCPCH,^1,3^ Vanessa Saliba, MD.^1^**

**1 Public Health England Colindale, 61 Colindale Avenue, London NW9 5EQ, UK,**

**2. London School of Hygiene and Tropical Medicine, London, UK**

**3. Paediatric Infectious Diseases Research Group, St. George’s University of London, London, UK**

**SUPPLEMENT MATERIAL**

Supplement Table S1: Size and number of bubbles in nurseries reporting a COVID-19 outbreak to Public Health England (PHE)

|  | **Total number of bubbles in setting** | | | | | | | | | | | **Total** |
| --- | --- | --- | --- | --- | --- | --- | --- | --- | --- | --- | --- | --- |
|  | **1** | **2** | **3** | **4** | **5** | **6** | **7** | **8** | **9** | **10** |  | |
| **Number of nurseries** | 13 | 42 | 56 | 29 | 11 | 6 | 5 | 3 | 4 | 2 | 171 | |
| **1 bubble only** | 13 (100%) | 11 (26%) | 11 (20%) | 6 (21%) | 2 (18%) | 0 (0%) | 0 (0%) | 0 (0%) | 0 (0%) | 0 (0%) | 43 (25%) | |
| **2 bubbles** | 0 (0%) | 31 (74%) | 11 (20%) | 5 (17%) | 4 (36%) | 1 (17%) | 2 (40%) | 1 (33%) | 0 (0%) | 1 (50%) | 56 (33%) | |
| **3 or more bubbles** | 0 (0%) | 0 (0%) | 34 (61%) | 18 (62%) | 5 (45%) | 5 (83%) | 3 (60%) | 2 (67%) | 4 (100%) | 1 (50%) | 72 (42%) | |

Supplement Table S2: Reported social distancing between and within bubbles in nurseries reporting a COVID-19 outbreak to Public Health England (PHE)

|  | **Within bubbles - Social distancing between:** | | |
| --- | --- | --- | --- |
|  | **Staff** | **Children** | **Staff and children** |
| **All the time** | 19 (11%) | 2 (1%) | 1 (1%) |
| **Most of the time** | 85 (49%) | 3 (2%) | 3 (2%) |
| **Some of the time** | 37 (22%) | 11 (6%) | 15 (9%) |
| **Rarely** | 15 (9%) | 29 (17%) | 42 (25%) |
| **Never** | 16 (9%) | 127 (74%) | 110 (64%) |
| Total | 172 | 172 | 171 |
|  |  |  |  |
|  | **Between bubbles - Social distancing between:** | | |
|  | **Staff** | **Children** | **Staff and children** |
| **All the time** | 83 (50%) | 102 (61%) | 85 (51%) |
| **Most of the time** | 64 (39%) | 40 (24%) | 44 (27%) |
| **Some of the time** | 14 (8%) | 6 (4%) | 15 (9%) |
| **Rarely** | 2 (1%) | 4 (2%) | 6 (4%) |
| **Never** | 3 (2%) | 14 (8%) | 16 (10%) |
| Total | 166 | 166 | 166 |
|  |  |  |  |

Supplement Table S3: Shared facilities in nurseries reporting a COVID-19 outbreak to Public Health England (PHE)

| **Facility** | | **Was the facility assigned per bubble or shared?** | | |
| --- | --- | --- | --- | --- |
|  |  | **Per bubble** | **Shared** | **Total** |
| Staff room | Number of Nurseries | 59 (35%) | 111 (65%) | 170 |
|  | Total number of cases (staff and children) | 392/1323 (30%) | 744/2358 (32%) | 1136/3681 (31%) |
|  | Median (IQR) | 7 (5-13) | 8 (4-12) | 8 (5-12) |
|  | Mode (Range) | 6 (2-33) | 3 (2-31) | 6 (2-33) |
| Staff bathroom | Number of Nurseries | 38 (22%) | 131 (78%) | 169 |
|  | Total number of cases (staff and children) | 280/810 (35%) | 850/2838 (30%) | 1130/3648 (31%) |
|  | Median (IQR) | 10.5 (5-12) | 8 (5-14) | 8 (5-12) |
|  | Mode (range) | 3 (2-33) | 6 (2-31) | 6 (2-33) |
| Children's eating space | Number of Nurseries | 146 (86%) | 23 (14%) | 169 |
|  | Total number of cases (staff and children) | 455/13021 (3%) | 48/1313 (4%) | 503/14334 (4%) |
|  | Median (IQR) | 8.5 (5-13) | 7 (4-9) | 8 (5-12) |
|  | Mode | 6 (2-33) | 2 (2-23) | 6 (2-33) |
| Children's bathroom | Number of Nurseries | 112 (67%) | 56 (33%) | 168 |
|  | Total number of cases (staff and children) | 361/9634 (4%) | 139/4592 (3%) | 500/14226 (4%) |
|  | Median (IQR) | 9 (5-13.5) | 7 (4-11.5) | 8 (5-13) |
|  | Mode | 12 (2-33) | 2 (2-31) | 6 (2-33) |

Supplement table S4: reason for testing in the first four cases, where known, among staff and children in nurseries reporting a COVID-19 outbreak to Public Health England (PHE)

| **Reason for Testing** | **Total** |
| --- | --- |
| Contact of community case | 3 (0.5%) |
| Contact of household case | 58 (9.6%) |
| Contact of school case | 92 (15.3%) |
| Other / not known | 39 (6.5%) |
| Symptomatic | 411 (68.2%) |
| **Total** | **603** |
